# Global Dam Tracker: A database of more than 35,000 dams with location, catchment, and attribute information

**DOI:** 10.1038/s41597-023-02008-2

**Published:** 2023-02-23

**Authors:** Alice Tianbo Zhang, Vincent Xinyi Gu

**Affiliations:** 1 Washington and Lee University, Department of Economics, Lexington, Virginia 24450 USA; 2 Georgia Institute of Technology, School of Public Policy, Atlanta, Georgia 30332 USA

**Keywords:** Environmental impact, Hydroelectricity, Energy policy

## Abstract

We present one of the most comprehensive geo-referenced global dam databases to date. The Global Dam Tracker (GDAT) contains 35,000 dams with cross-validated geo-coordinates, satellite-derived catchment areas, and detailed attribute information. Combining GDAT with fine-scaled satellite data spanning three decades, we demonstrate how GDAT improves upon existing databases to enable the inter-temporal analysis of the costs and benefits of dam construction on a global scale. Our findings show that over the past three decades, dams have contributed to a dramatic increase in global surface water coverage, especially in developing countries in Asia and South America. This is an important step toward a more systematic understanding of the worldwide impact of dams on local communities. By filling in the data gap, GDAT would help inform a more sustainable and equitable approach to energy access and economic development.

## Background & Summary

As one of the oldest forms of man-made infrastructure, dams have been integral to economic development throughout human history. They are built to control floods, irrigate crops, supply water, generate electricity, and ease navigation. Proponents of dams often praise them as a source of low-carbon electricity. Hydropower generated 16 percent of the world’s total electricity and 60 percent of all renewable electricity in 2019^1,2^. Estimated to reduce annual emissions by about 2.8 billion tons of carbon dioxide equivalent (CO_2_e), hydropower is often seen as the backbone for achieving the goals of affordable and clean energy for all (SDG 7). Developing countries in Asia, South America, and Africa possess significant untapped hydropower potential^3^. Globally, at least 3,700 large hydropower plants with a capacity of more than 1 MW and 82,800 smaller plants are operating, under construction, or being planned^4,5^.

But harnessing the power of the river comes with concentrated costs. The construction of dams, especially large dams and those located in transboundary river basins, has wide-ranging socioeconomic, geopolitical, and environmental implications. The International Commission on Large Dams (ICOLD) defines a large dam as “a dam with a height of 15 metres or greater from lowest foundation to crest or a dam between 5 metres and 15 metres impounding more than 3 million cubic metre.” Large dams, such as the Three Gorges Dam in China, displace millions of people globally and deprive entire communities of their cultural heritage^6,7^. The displaced rarely receive adequate compensation and often suffer from an enduring loss of land, job, and wealth long after the dams are built^8^. By modifying and fragmenting rivers, dams also exacerbate water scarcity^9–11^ and worsen geopolitical tensions^12,13^. As these socioeconomic and geopolitical costs are often borne by marginalized and indigenous people^14^ near the dam catchment area, dams could exacerbate existing social and environmental injustice.

Beyond the socioeconomic and geopolitical implications, dams affect the ecological functioning of river systems by reducing the downstream transfer of nutrients^15^ and threatening the natural habitat of freshwater megafauna^16^, especially migratory fish species^17,18^. A third of the world’s freshwater fish species are at risk due to dam construction booms in some of the most biodiverse river basins, such as the Amazon, Congo, and Mekong^19,20^. In addition, some dams may intensify rather than mitigate global warming, as these man-made waterbodies reduce surface albedo and result in a positive radiative forcing^21^. Climate change mitigation requires a rapid and systematic transformation of the energy system toward renewable sources. As such, understanding the costs and benefits of dam construction is crucial for energy and climate policy.

Quantifying the net benefit of dams requires a comprehensive database with three key characteristics: 1) accurate geo-coordinates of dams, 2) information on completion year, and 3) global in coverage. Existing databases of dams either lack necessary spatial and temporal detail or are limited in scope. Two recently published global dam databases, GOODD^22^ and GeoDAR^23^, make significant contributions to geocode and validate dam and reservoir locations. However, neither database contain publicly available dam attribute information, limiting their use for inter-temporal studies. To fill this gap, we compile a new global database of dams, the Global Dam Tracker (GDAT), by consolidating existing global dam datasets with country- and region-specific data from nonprofit organizations, academics, and governments^24^. Building upon existing global and regional dam databases, including AQUASTAT, the Global Reservoir and Dam Database (GRanD), and the World Resources Institute (WRI) database, we conduct extensive cross-referencing and manual validation to fill in data gaps and correct erroneous dam attributes. More than 90% of dams in GDAT are geocoded (n = 31,780), and the coordinates are extensively verified using Google Earth and other geospatial software. Beyond location, GDAT contains detailed attribute information for each dam, such as completion year, purpose, height, length, and installed capacity. As such, GDAT is one of the most comprehensive geo-referenced global dam databases with catchment and attribute information to date, especially for the Global South.

To allow for inter-temporal analysis of the impact of dam construction, we develop an algorithm that uses GDAT and various state-of-the-art satellite data products to obtain reservoir and catchment areas associated with dams in GDAT. To demonstrate the use case of GDAT data, we calculate the cumulative change in global surface water from dam construction and find that dams have substantially altered the location and persistence of surface water around the world. Our results show a cumulative increase of approximately 50,000 km^2^ of seasonal and permanent water in dam catchment areas between 1984 and 2018.

The GDAT database could be used for a systematic and global analysis of the impact of dams on local communities. As the urgency of climate change calls for a transition from fossil fuels, hydropower could usher in a cleaner and more sustainable energy system as mandated by SDG 7. But these benefits should be weighed against the negative social, geopolitical, and environmental costs. Therefore, a global dam database that improves our understanding of the net impact of dams is crucial for informing a more sustainable and equitable approach to economic development.

## Methods

In this section, we present an overview of the data collection process and demonstrate an application where GDAT is used to calculate dam-induced global surface water changes. The first subsection, “Global Dam Tracker (GDAT),” presents the GDAT database and describes the data sources. The second subsection,“GDAT Use Case,” details our application of GDAT and outlines an algorithm for calculating surface water changes within dam catchment areas.

### Global dam tracker (GDAT)

The Global Dam Tracker (GDAT), a database with 35,140 dams in all continents except Antarctica, is an original data compilation effort^24^. We build upon existing global and regional dam databases, including AQUASTAT (www.fao.org/aquastat), the Global Reservoir and Dam Database (GRanD - globaldamwatch.org/grand)^25^, and the World Resources Institute (WRI) database (datasets.wri.org/dataset/globalpowerplantdatabase)^26^. For each country, we consolidate these global and region databases with available data from government agencies, academics, and nonprofits. More than 90% of dams in GDAT are geocoded (n = 31,780), and the coordinates are extensively verified using Google Earth and other geospatial software. This process creates an extensive cross-validated database that can be used for analysis at various geographical scales.

#### Description of regional data sources

This section provides an overview of major regional or national data sources that contributed substantially to GDAT. Detailed information on the data sources for each country is available in the Region Highlights section. Existing dam databases have limited coverage of developing and low-income countries. As such, our data collection effort focuses on countries in Africa, Asia, and South America, where we collected primary data from administrative and other sources. Data for countries in Europe, North America, and Oceania are mainly obtained from existing global dam databases.

Dams in India were collected from the India Water Resources Information System (WRIS), a database that is maintained and funded by the government of India (indiawris.gov.in/wris). Reservoir volume data are not provided by the WRIS and, hence, are not currently available in GDAT for dams in India.

Dams in China were compiled from several nongovernmental sources: AQUASTAT, GRanD, International Rivers, and the Consultative Group for International Agricultural Research (CGIAR). While the Ministry of Water Resources of the People’s Republic of China maintains a database on dams in China, it is not released to the public. Hence, even though the GDAT database has compiled more than 1,000 dams in China, the actual dam count is expected to be much higher, given that many smaller dams might be missing from the database.

For Continental Southeast Asia, GDAT database builds upon three main data sources: AQUASTAT, GRanD, and CGIAR Greater Mekong (wle-mekong.cgiar.org). The CGIAR database focuses on the major river basins of continental Southeast Asia (Mekong, Red, Salween, Irrawaddy) and contains information for more than 800 dams that span China, Thailand, Vietnam, Laos, Cambodia, and Myanmar.

Dams in the Middle East came from AQUASTAT and GRanD, except Turkey and Iran, where we obtained data from government websites. The Turkish General Directorate of State Hydraulic Works (www.dsi.gov.tr)publishes information for more than 600 dams, and the Iran Water Resources Management Company (www.wrm.ir) publishes information for more than 1,000. Neither database, however, is geolocated. Hence, the geographic coordinates for these two countries are manually verified through GeoNames and Google searches.

Dams in Brazil were compiled from four sources: AQUASTAT, GRanD, the Brazilian National Dam Safety Information System (SNISB), and Dams in Amazonia (archive.internationalrivers.org). The SNISB for 2016, the most recent version available, includes almost 23,000 dams. We excluded all dams and reservoirs used exclusively for tailings and hazardous waste storage. The SNISB includes structures with a height greater than or equal to 15 meters and a total reservoir capacity greater than 3hm^3^. As a result, the GDAT database is likely missing a considerable number of small dams in Brazil that are not monitored by the SNISB.

While most of the new data in GDAT came from the developing world, especially Africa, Asia, and South America, we also cleaned and consolidated data for Europe, Oceania, and North America from existing global databases and government sources. Data for Oceania mostly came from AQUASTAT and GRanD, except for Australia, which was collected from the Register of Large Dams maintained by the Australian National Committee on Large Dams. The GDAT database for Europe contains information from AQUASTAT, GRanD, the WRI Global Power Plant Database, and reports for more than 3,000 dams in Europe. For North America, data were compiled from AQUASTAT and GRanD, except for the United States, which included data from the United States Geological Survey (USGS). USGS lists more than 8,000 dams, dikes, levees, and other water-engineering structures. We filtered the USGS data to remove non-dam structures.

#### Description of dam attributes

In addition to greater spatial coverage, the GDAT database contains detailed attribute information for each dam. These attributes include basic identifying information such as the dam name and alternate names; geographic attributes such as country, province, river basin, latitude, and longitude; dam characteristics such as completion and construction years, height, and length; reservoir capacity and area; purposes; and energy-generating capacity for hydroelectric dams. These attributes served as the basis for data collection, and any information found in regional and country-specific sources was matched to these attributes to be included in GDAT in a consistent and standardized format.

**Fig. 1:**
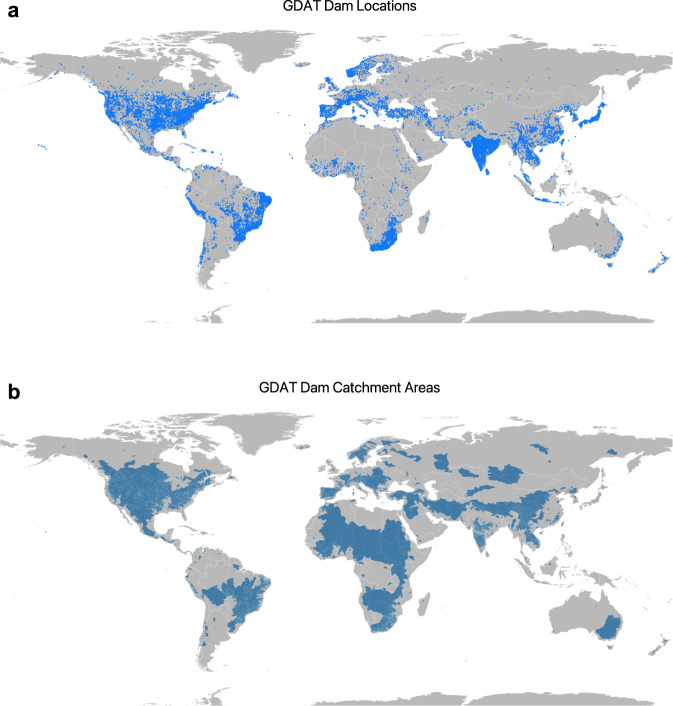
Map of dams in the Global Dam Tracker database. (**a**) Locations of all dams in the GDAT database, with each dam represented by a blue point. Large concentrations of dams can be found in the United States, Brazil, India, South Africa, Europe, and East and Southeast Asia. (**b**) The catchment area of dams delineated using drainage flow directions.

Dams are largely concentrated along major river basins in Asia, Africa, Europe, and the Americas (Fig. 1 and Table 1). Asia has the highest number of dams completed to date, with 9,526 dams, or 27 percent of worldwide dam construction. North America and South America also have significant dam infrastructure, representing 23 percent and 21 percent of the global dam count, respectively (Table 1). In terms of installed capacity, Asia and South America account for 50 percent and 20 percent of the global total installed capacity, respectively, while Europe and North America account for 18 percent and 9 percent, respectively (Table 1).

**Table 1:** Summary statistics of dam attributes in GDAT.

	Continent						World
	Asia	North America	South America	Africa	Europe	Oceania	
**Total dam count**	9,526	8,333	7,566	6,243	3,201	271	35,140
**Count by main purpose**							
Irrigation dams	5,627	1,130	1,607	253	241	—	8,858
Hydropower dams	1,255	1,553	1,372	345	2,458	121	7,104
Water supply dams	395	1,147	1,118	149	225	136	3,170
Flood control dams	390	1,748	53	18	63	—	2,272
Recreation dams	4	1,323	114	—	8	—	1,449
Other/not specified dams	1,819	1,139	2,894	5,223	198	14	11,287
**Key dam attributes**							
Median completion year	1981	1963	1990	1981	1966	1966	1974
Mean dam height (m)	33	25	18	14	57	37	26
Mean dam length (m)	1,014	1,335	817	391	1,129	No data	1,036
Total installed capacity (MW)	468,681	85,173	193,496	No data	175,258	4,553	927,161
Total reservoir volume (km^3^)	1,091	13,477	1,179	No data	1,392	17	17,155
Total reservoir area (km^2^)	43,264	316,058	10,501	No data	146,772	1,169	517,763

**Fig. 2:**
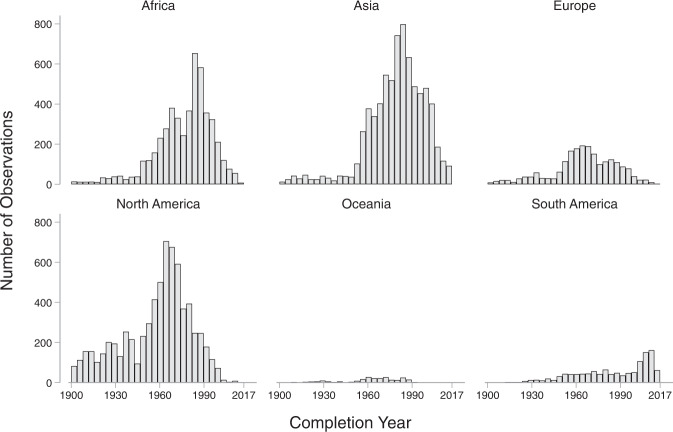
Distribution of dam completion year by continent.

**Fig. 3:**
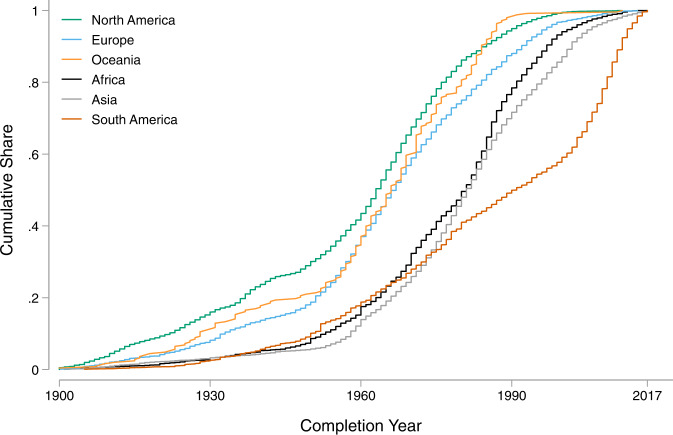
Cumulative distribution of dam completion year by continent.

There are notable differences in the distribution of dam completion years across continents. While most developed countries in North America, Europe, and Oceania have been witnessing a decline in dam construction since the 1970s, developing countries in Africa, Asia, and South America have been experiencing a continued increase in dam construction (Fig. 2). Most notably, the Yangtze basin in China, the Ganges-Brahmaputra basin in South Asia, and the Amazon basin in South America have many dams currently planned or under construction^4^. A time series of completion year (Fig. 3) demonstrates a significant acceleration in dam construction from 1970 to 2000, followed by a slight deceleration over the past decade. Global investments in hydropower peaked in the second half of the twentieth century in response to a growing desire to diversify energy sources and reduce dependency on fossil fuels^5^.

**Fig. 4:**
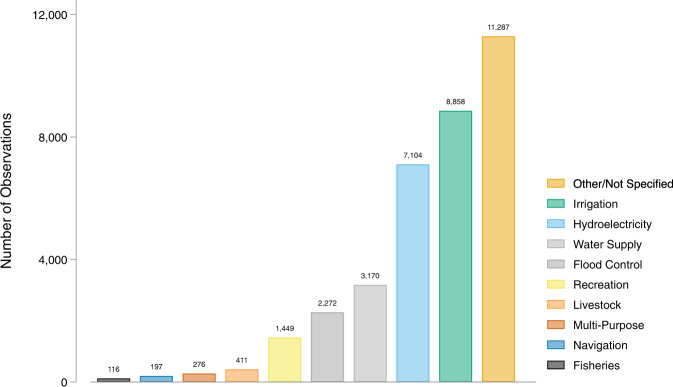
Distribution of main purpose of dams in GDAT – Overall.

The most common main purposes of dams in GDAT are irrigation and hydroelectricity, which represent 25 percent and 20 percent of the data (Fig. 4). Additional main purposes include water supply, flood control, recreation, livestock, and navigation. 32 percent of dams had the main purpose classified as “other” due to limited or missing information in the primary sources.

**Fig. 5:**
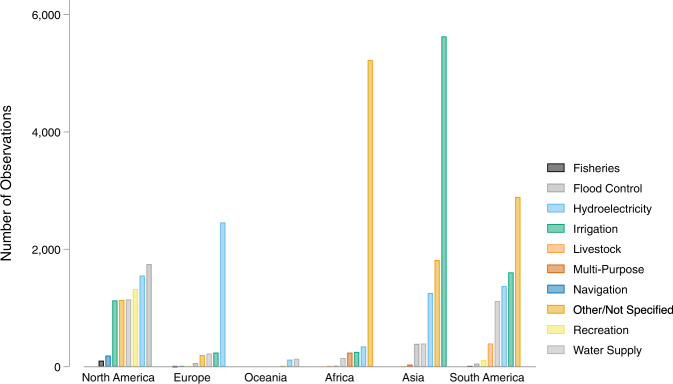
Distribution of main purpose of dams in GDAT – By continent.

In Asia and South America, irrigation is the most common main purpose of dams (Fig. 5). In comparison, the most common main purposes in North America, Europe, and Oceania are flood control, hydroelectricity, and water supply/storage, respectively. The number of dams shown under each main purpose reflects the primary and secondary sources used in the data collection. For example, Europe’s high percentage of dams used for hydroelectricity is partly due to the high concentration of hydroelectric power plants in the WRI data. Additional sources will need to be consulted to provide a more complete representation of the dam main purposes by region, particularly for Europe and Oceania.

### GDAT Use Case: Calculating Dam-Induced Global Surface Water Changes

**Fig. 6:**
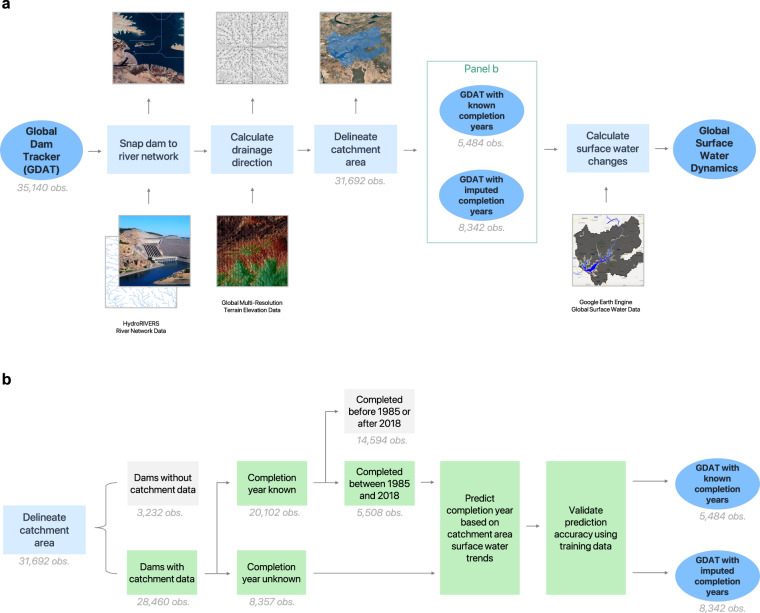
Conceptual diagram of the algorithm. We develop an algorithm to estimate the changes in global surface water from dam construction. (**a**) To calculate the difference in surface water coverage before and after dams are built, we snap each dam to a river network to correct for its location, calculate the drainage direction, and delineate its catchment area. (**b**) Procedures for imputing the completion years of dams when the information is missing. This allows the analysis of surface-water changes to be expanded beyond dams with known completion years.

To demonstrate how GDAT would improve our understanding of the global impact of dam construction, we use GDAT to calculate dam-induced global surface water changes over the past three decades. Following prior work^22,27^, we apply an algorithm (Fig. 6a) that uses GDAT and various state-of-the-art satellite data products to analyze temporal surface water dynamics around dam catchment areas. The algorithm has five main steps.

First, to ensure that dams fall precisely on a river network, we snap all 31,780 geocoded dams in GDAT onto the closest river lines using a fine-scale global river network dataset at 15 arc-second resolution, HydroRivers. HydroRivers provides vectorized line network of all global rivers that have a catchment area of at least 10 km^2^ or an average river flow of 0.1 cubic meters per second, or both. It encompasses 8.5 million individual river reaches with an average length of 4.2 km, representing 35.9 million km of rivers globally^27^. This step ensures the dam coordinates are exactly aligned with the river network.

Second, we use the USGS Global Multi-Resolution Terrain Elevation 2010 data (GMTED 2010) at 7.5 arc-second resolution to generate the drainage flow direction at each dam site. GMTED 2010, developed by the USGS and the National Geospatial-Intelligence Agency (NGA), is an enhanced global elevation data of choice that incorporates the best available data sources^28^. The GMTED 2010 data represent a significant improvement in consistency and vertical accuracy over existing global elevation data, such as GTOPO30.

Third, with the PCRaster library in Python, we delineate the catchment area representing the buffer zone upstream of the dam location containing its reservoir. The PCRaster code assigns a flow direction value to each pixel in the GMTED elevation data, which it does by analyzing the values of the eight surrounding pixels. The code then retraces the flow of water starting from each dam by moving upstream until there are no longer any more upstream pixels or until the algorithm hits another dam^29^. The area covered by the retraced pixels is the catchment area for the dam, which is the area we use to calculate surface water changes caused by the dam. In total, we obtain 28,460 catchment areas (Fig. 1b) corresponding to dams in GDAT. A small subset of GDAT dams does not have catchment areas due to limitations in the elevation and river network data.

**Fig. 7:**
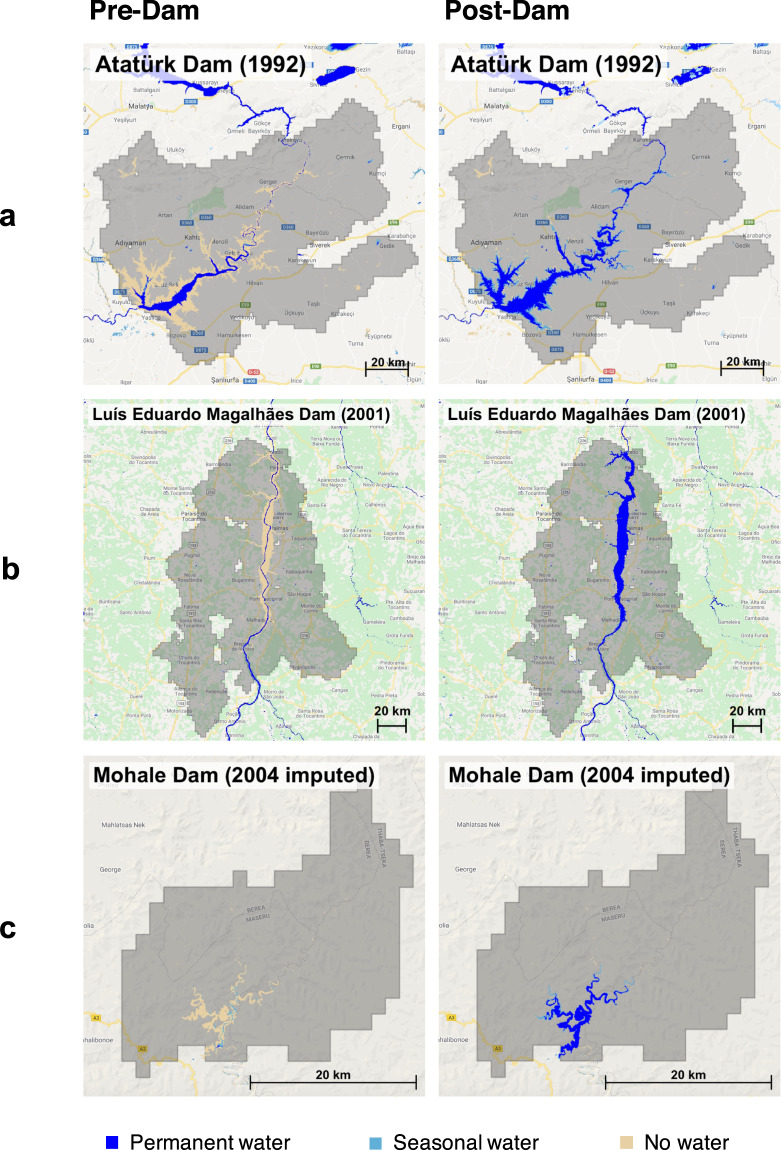
Change in surface water coverage for three exemplary dams. Dam completion year in parenthesis. (**a**) Construction for Ataturk Dam in Turkey was completed in 1990 and the reservoir was filled in 1992. A comparison of the pre-dam (left) and post-dam (right) images show a clear increase in water pixels after the completion, a total increase of 983,482 km^2^. The pre-dam image exhibits a partially filled reservoir, demonstrating that large dams may take an extended time to fill. (**b**) Luis Eduardo Magalhaes Dam in Brazil was completed in 2001. The formerly free-flowing section of the river is visible in the pre-dam image, while the dam flooded an area of 660,130.5 km^2^. (**c**) Mohale Dam in Lesotho, with an imputed completion year of 2004, illustrates the logic behind our imputation procedure. The actual year of construction is 2002, and the dam was formally commissioned in 2003–4. After completion, the dam flooded an area of 15,884.5 km^2^.

Calculating surface water change requires information on the dam completion year. Since more than 70% of GDAT dams have known completion years, we use these as training data for an imputation procedure on dams with missing information (Fig. 6b). Around the year of dam completion, we expect to see a significant change in the upstream catchment area, such as in Fig. 7. Therefore, as the fourth step, we impute the completion year for 8,342 dams by detecting trend breaks in the catchment area time series. The satellite-derived surface water coverage between 1984 and 2019 comes from the Global Surface Water (GSW) V1.1 dataset available through Google Earth Engine. Developed by the European Commission Joint Research Centre, GSW is a globally consistent, validated dataset that shows the spatial and temporal distribution of global surface water over the past three decades using three million Landsat satellite images^30^.

In the fifth and final step, we calculate global surface water changes induced by dam construction. From GSW, we obtain pixel counts for the “no water,” “seasonal water,” and “permanent water” categories as annual time series. We then use the completion year information to calculate the median pre- and post-dam pixel counts. We choose the median as the summary measure to minimize idiosyncratic measurement errors from climatic or technical factors that may affect satellite image quality. We quantify the temporal surface water dynamics for 11,710 dams completed between 1985 and 2019. We drop dams without pre- or post-completion observation, i.e., those built before 1984 or after 2019. Below, we provide additional details about the methodology.

#### Dam catchment areas

We combine GDAT with elevation data to calculate the catchment area that contains the reservoir of each dam. To trace the drainage flow direction, we use GMTED 2010 with a resolution of 250 meters. The elevation data allows us to calculate how water would flow between each pixel based on their relative upstream or downstream location, similar to GOODD^22^. We first employ PCRaster, an environmental modeling software, to obtain catchment areas for each dam from elevation data^29^. We use the local drainage direction function (ldd) to assign flow directions to each pixel based on the lowest elevation of the eight surrounding pixels. This allows us to obtain a flow-direction file from elevation data. We then use the subcatchment function, which uses each dam in GDAT as a starting point and traces all possible trajectories that water could flow toward the dam. If there is another dam along the path of the water trajectory, the function stops, as all further upstream points would fall within another dam’s catchment area. We are hence able to capture all upstream points that eventually drain to the point of each dam, enabling us to obtain the catchment areas for the majority of dams in our database.

Data on flow direction contains noise from the slopes surrounding the river, as water might flow in a different direction than the river itself^22^. The pixels where the greatest flow accumulation occur (the path tracing the bottom of the riverbed) are the only locations that accurately reflect the river’s flow. As a result, dams that are even slightly misplaced relative to the main river path could result in large errors in the catchment area. To address this, we align dam locations with river network data from HydroRIVERS to ensure that dams fall precisely on the main river path where flow accumulation occurs. HydroRIVERS captures all pixels with an accumulated upstream catchment of more than 10 km^2^, producing a connected network of lines representing accumulated river flows^31^. We use the interpolate function from the Shapely library in Python to snap the location of each dam onto its nearest river network^32^.

The vast majority of dams in the GDAT database are located very close to a HydroRIVERS river network line, with 30,471 dams (or 95.9 percent) falling within 0.5 degrees of their snapped location. The remaining 1,309 dams were snapped at a distance of more than 0.5 degrees. Some of them were located on islands not covered by HydroRIVERS, causing them to be snapped onto a continent. Hence, dams with an interpolated distance of greater than 0.5 degrees may be inaccurate. This is due to limitations in the data resolution, as 0.5 degrees is the resolution of the runoff and discharge layers used to calculate HydroSHEDS^33^. In those cases, we discard the snapped location and instead use the original geo-coordinates. Our final catchment-area calculation procedure, therefore, consists of three stages: obtaining drainage direction from elevation data via the ldd function from PCRaster, snapping each dam onto its respective river network via Shapely, and using the drainage direction and interpolated location to derive a catchment area for each dam. The output is a raster file of dam catchment areas, which are used to understand how each dam contributed to changes in surface-water coverage.

#### Calculating surface water changes within dam catchment areas

We calculate the change in surface water coverage within each catchment before and after dam completion. This allows us to understand how the dam has inundated the surrounding land. We use data from the Global Surface Water (GSW) Explorer developed by the European Commission Joint Research Centre, which contains surface water coverage data between 1984 and 2019^30^. The dataset divides the world into pixels coded as 1 for no coverage, 2 for seasonal coverage, and 3 for annual coverage. We mask all pixels that are coded as 0 (no data). Not all years contain robust data due to cloud cover and satellite-imaging anomalies. Therefore, for each dam, we obtain the median value of all pre-dam pixels within its catchment area and create a single snapshot of the median pre-dam water coverage. We do the same for the post-dam pixels (inclusive of the year of completion), obtaining a single post-dam snapshot of the median water coverage. Due to the masking of no-data pixels, some pixels may have fewer years of data. The possible median-value pixels include no water (1), varying values of seasonal water (1.5, 2, 2.5), and permanent water (3). Once we have the median-value snapshots of pre-dam and post-dam water coverage within the buffer zone of each dam, we calculate the difference in the amount of area covered by both seasonal and permanent pixels. Given that GSW data spans 1984 and 2019, pre- and post-dam comparisons are only possible for dams with catchment data and completion years that fall within the GSW time range. This yields us 5,508 dams for which we can calculate water-coverage changes.

#### Imputation of dam completion years

Calculating surface water changes using dams with available completion-year data limits us to only 5,508 dams out of more than 30,000 in GDAT. To expand our analysis to more dams, we developed a machine-driven imputation procedure that calculates the dam’s completion year based on surface-water data from GSW. Because the imputation procedure requires deriving completion years from surface-water changes, we can only apply it to dams with catchment data but without recorded completion years. This totals 8,357 observations. Using GSW data, we obtain the annual pixel counts within the catchment area of each dam for each year between 1984 and 2019. The result of this step is a data frame of 8,357 dams spanning 36 years, with counts of no-water (1), seasonal-water (2), and permanent-water (3) pixels for each year. Years that contain missing data are filled in with data from the previous year or the most recent year with data.

We use two methods to calculate the imputed completion year. The first method identifies the year with the largest pixel change as the imputed completion year. We conduct four runs for this method: using 1 pixels only (no water), 2 pixels only (seasonal water), 3 pixels only (permanent water), and 2 + 3 pixels (any water). For each run, we obtain the pixel difference between the current and prior years for each year within the GSW data, which yields 35 observations of year-to-year differences. This yields us a total of four imputation results using the largest-change method. The second method involves the calculation of the structural break using the Wald test. A structural break occurs when the trend of a time series abruptly changes^34–36^. We apply the STATA estat sbsingle test, which assumes a single structural break with an unknown break date in the data^37–40^. This is appropriate for estimating the completion year of a dam when the year is unknown, given that the structural break in the water-coverage trend would occur when the dam is completed. As with the first method, we apply the structural-break test with four runs: 1 pixel only (no water), 2 pixels only (seasonal water), 3 pixels only (permanent water), and 2 + 3 pixels (any water). This yields four more imputation results for each dam. The above two methods combined yield a total of eight imputation results. The most frequent (mode) year among the eight imputations is the final calculation for the imputed year. If there are multiple modes, then the earliest mode is taken. Taking earlier years would account for the fact that it takes time for dams to fill up after completion.

#### Use case results: dam-induced global surface water changes

**Fig. 8:**
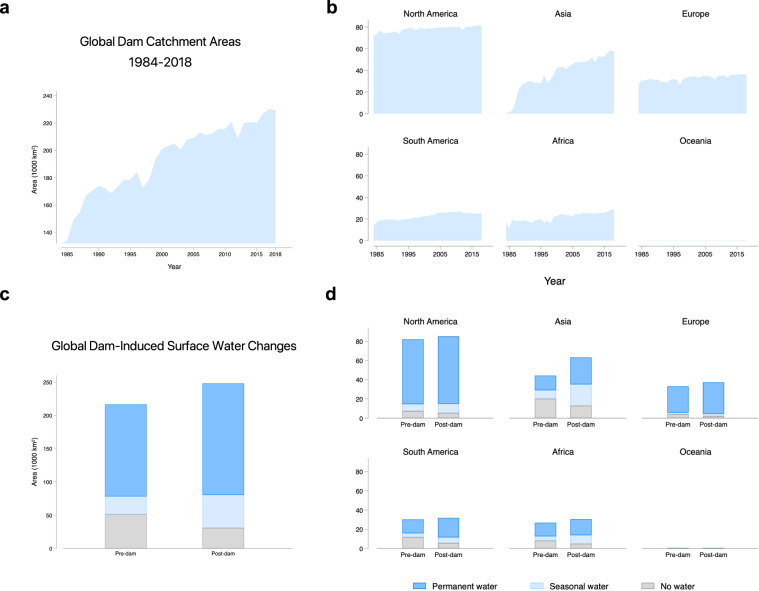
Summary of changes in water coverage around global dam catchments between 1984 and 2018. (**a**) Global cumulative increase in water-covered area (seasonal + permanent) within dam catchments. (**b**) Cumulative increase by continent. (**c**) Cumulative increase by pixel category for pre- and post-dam periods, showing an increase in permanent- and seasonal-water pixels by around 50,000 km^2^ and a decrease in no-water pixels. (**d**) Dam-induced surface water changes by continent.

Dams have substantially altered the location and persistence of surface water around the world. Figure 8a shows a cumulative increase of at least 49,715 km^2^ of seasonal and permanent water in dam catchment areas between 1984 and 2018. This is likely a lower-bound estimate due to the absence of satellite data before 1984 and the lack of completion year information for all dams. The fastest dam-induced surface water expansion has occurred in Asia (Fig. 8b), particularly from the rapid pace of dam construction in China and India. Growth is slower but still considerable in South America and Africa, where hydroelectric dams power economic development in Brazil, Venezuela, Zambia, and D.R. Congo. North America witnessed a large number of dams at the start of the sample period, but few new dams were completed after 1984 (Figs. 2,3). Similar to North America, dam construction peaked in Europe around the 1960s, and surface water coverage within dam catchment areas has remained steady since 1984.

Comparing the change in seasonal and permanent water before (pre-dam) and after (post-dam) dam completion (Fig. 8c,d) gives us insight into the impact of dam construction on surface water dynamics. In Asia, post-dam catchment areas witnessed the largest surface water increase by more than 23,400 km^2^ (Table 2). This is followed by South America and Africa, with more than 7,000 km^2^ of additional surface water due to dam construction. We also observe an increase in the total pixel count globally and a pronounced increase in Asia as the spatial and temporal resolutions of satellite data there have improved substantially over the past two decades. This is a data quality limitation for quantifying the long-term surface water dynamics using satellite data–a caveat documented in the GSW dataset^30^.

**Table 2:** Summary statistics of global surface water changes by continent.

Continent	Number of Dams	Surface Water Coverage (km^2^)		
		Pre-dam	Post-dam	Difference
Asia	3,115	24,279	47,775	23,496
South America	4,119	18,641	26,259	7,617
Africa	1,987	18,681	25,945	7,265
Europe	1,117	29,122	35,249	6,127
North America	1,352	74,684	79,795	5,111
Oceania	20	124	223	100
Total	11,710	165,530	215,245	49,715

**Fig. 9:**
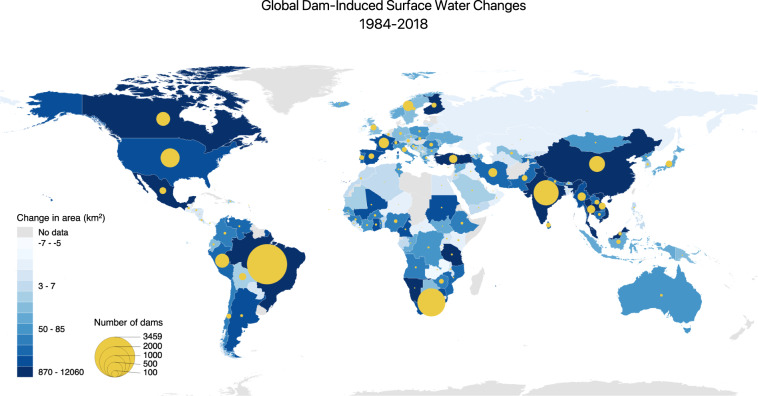
Surface water change in global dam catchments between 1984 and 2018 by country. The largest increase in surface water coverage are in developed and rapidly developing countries with significant dam counts, such as Brazil, Canada, China, India, the United States, Mexico, Turkey, and various Southeast Asian countries. The data captures changes in the median surface water pixel counts between pre- and post-construction periods within dam catchment areas. In addition to dam construction, a portion of the surface water changes in some countries, especially those in Asia, could be attributed to improvements in satellite data quality.

We map changes in global dam catchments by country to understand the spatial distribution of surface water dynamics (Fig. 9). The continental summaries mask considerable spatial heterogeneity across countries. The largest contributors to dam-induced surface water change come from developing countries with rapid hydropower installation, such as China and Brazil. China alone accounts for more than half of the total surface water increase in Asia. In Brazil, 3,459 dams contribute to more than 80% of the water expansion in South America. Developed countries with historically large inventories of dams, such as Canada and the United States, also see a significant rise in surface water. The expansion in surface water, however, may not be a universal feature of dams. In a few countries, most notably Zambia, Ireland, and the United Arab Emirates, surface water may have decreased slightly after dams are built. We cannot rule out the possibility that the decline is due to idiosyncratic changes in satellite data quality or measurement error, as we have found the surface water loss to be less than 5 km^2^ in these three countries. Echoing prior findings^30^, these results suggest that losses in permanent and seasonal water from dams may be more concentrated than gains.

## Data Records

The GDAT database is available as a zip folder (GDAT_v1.zip) on Zenodo^41^. Data records are provided as two ESRI shapefiles. The first file contains a point layer for dam locations (GDAT_v1_dams.shp). The second file contains a polygon layer for dam catchment areas (GDAT_v1_catchments.shp). Both shapefiles use the World Geodetic System 1984 (WGS84) datum. Dam attributes are available in both the point and polygon layers. In addition, a detailed codebook (GDAT_v1_documentation.xlsx) provides the definition of each variable in the attribute table.

## Technical Validation

### Comparison with existing datasets

#### Dams

**Fig. 10:**
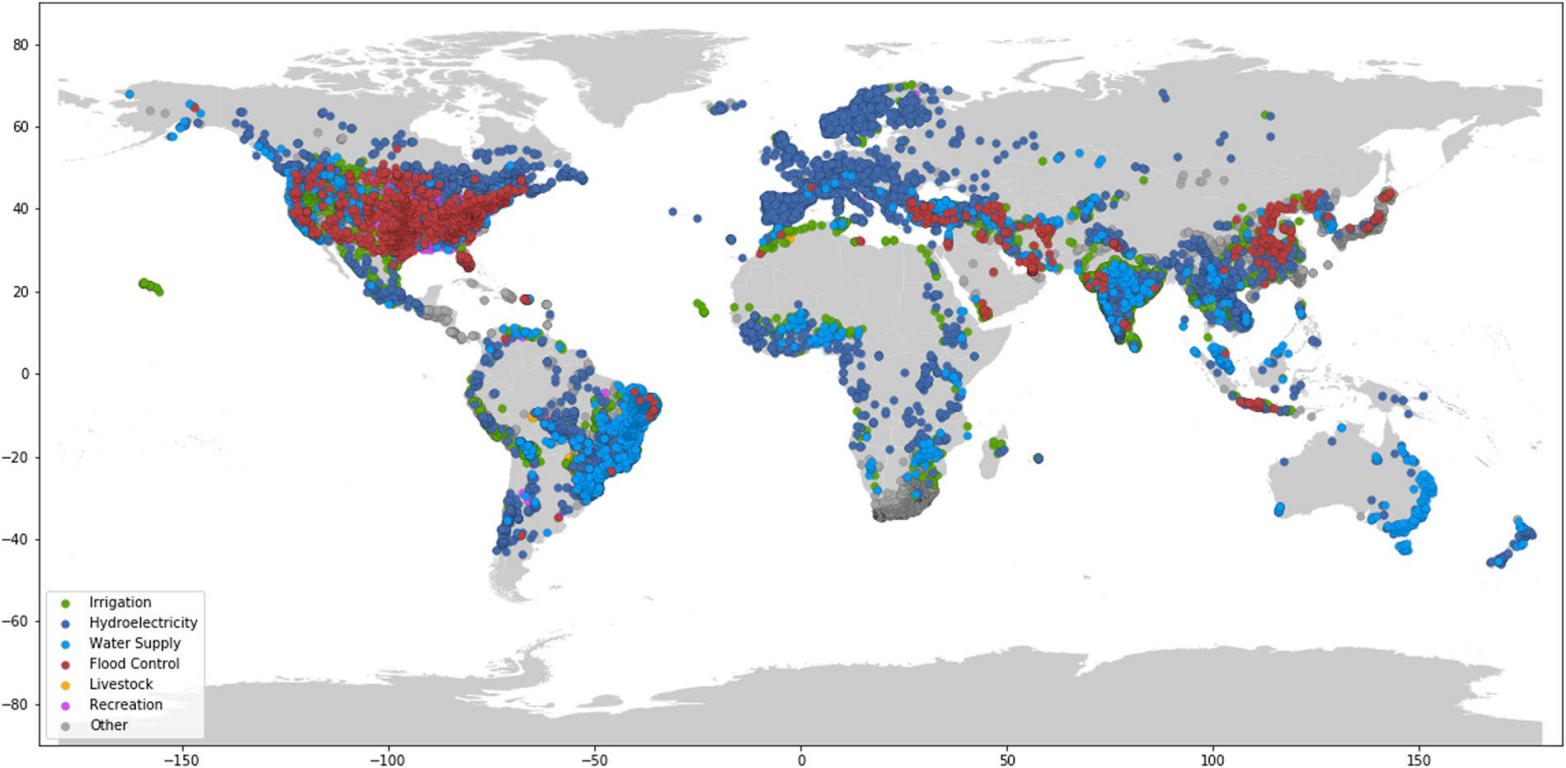
Map of Global Dam Tracker (GDAT) database – Global dams by location and main purpose.

**Fig. 11:**
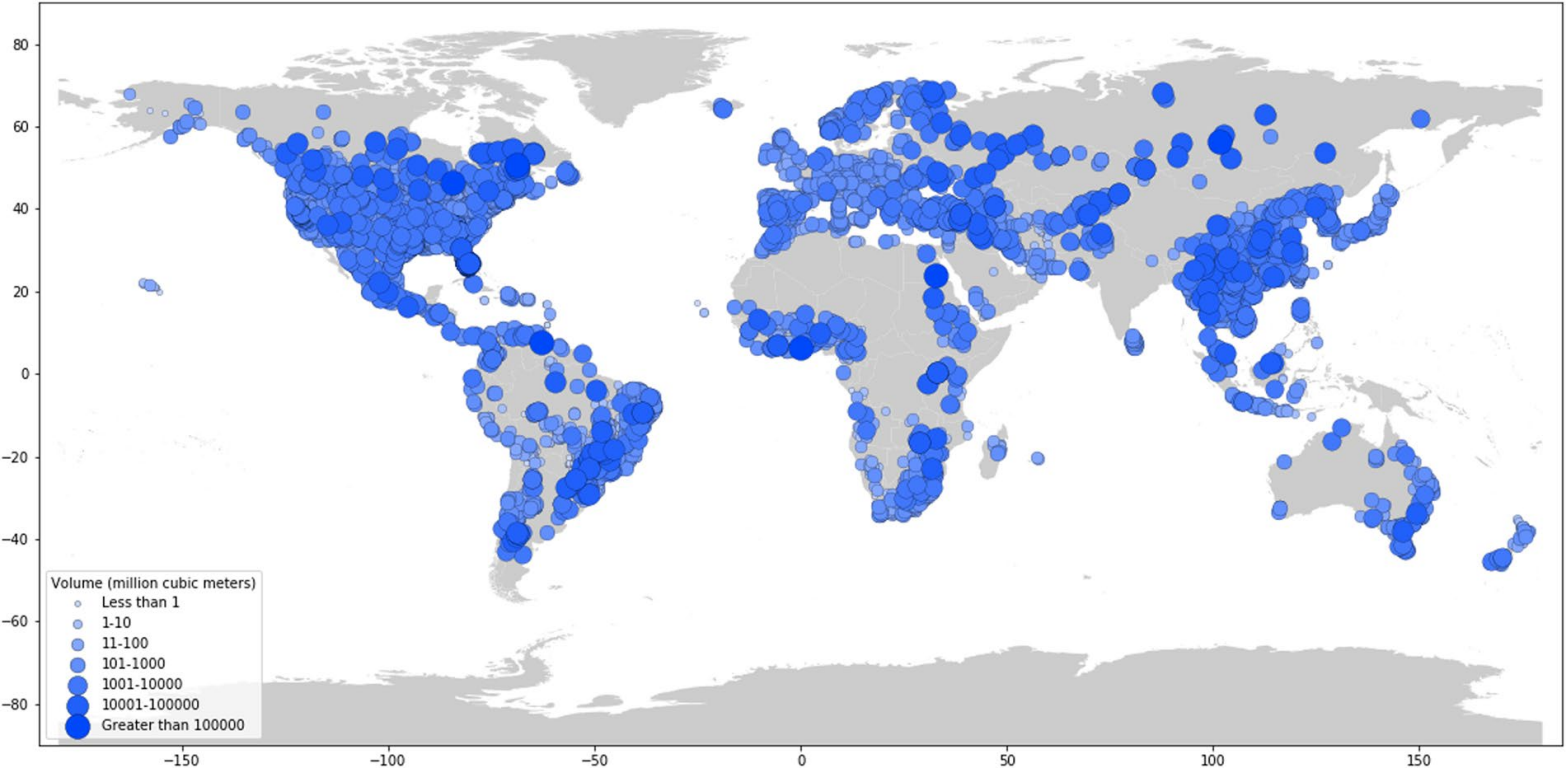
Map of Global Dam Tracker (GDAT) database – Global dams by reservoir capacity.

**Fig. 12:**
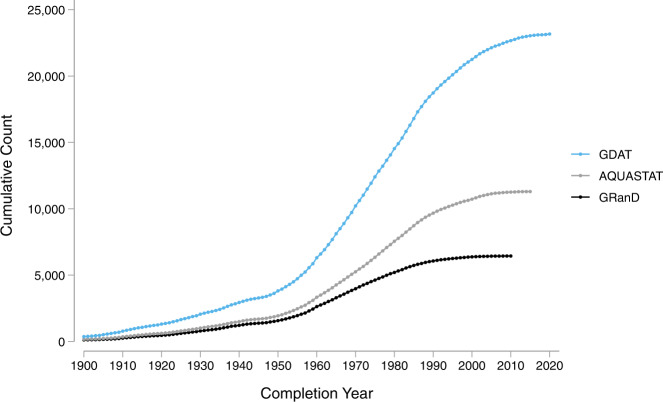
Cumulative count of dams by completion year – GDAT vs. other databases.

**Fig. 13:**
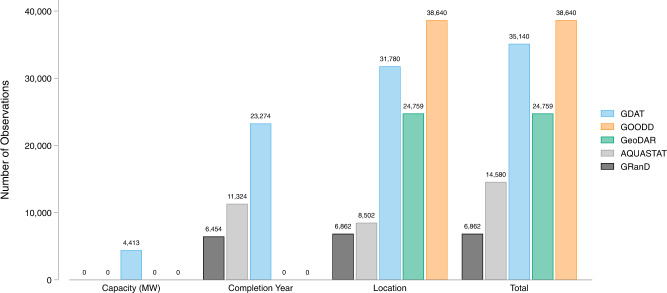
Comparison between GDAT and other databases – Availability of dam attributes.

We compare GDAT with four existing global dam databases, including GRanD, AQUASTAT, GOODD, and GeoDAR. We focus on GRanD and AQUASTAT when comparing attributes because GOODD and GeoDAR do not contain publicly available information on dam or reservoir attributes. As a result, this limits our ability to independently verify data accuracy and check for duplicates. The GDAT database (Figs. 10,11) surpasses GRanD, AQUASTAT, and GeoDAR not only in total dam count (Fig. 12) but also in attribute information on completion year, location, and generation capacity (Fig. 13). Specifically, GDAT contains 412 percent and 141 percent more records than the GRanD and AQUASTAT databases, respectively. GDAT also surpasses the total dam count of GeoDAR by 41 percent. For temporal coverage, GDAT contains data up to 2018, whereas the GRanD and AQUASTAT databases include dams constructed up to 2010 and 2013 (Fig. 12). The WRI’s Global Power Plant Database is excluded from this comparison because it focuses on power plants and, hence, contains mostly hydroelectric dams. For a visual comparison of geospatial coverage between the databases, please see the Region Highlights section below.

**Fig. 14:**
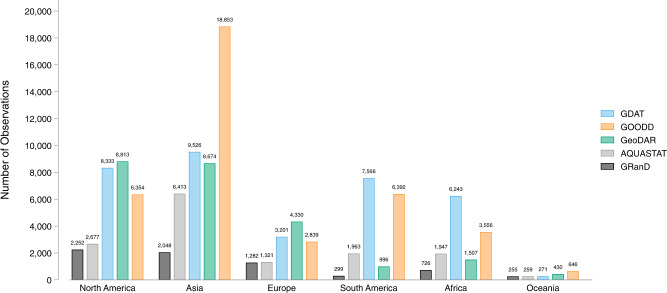
Comparison between GDAT and other databases – Dam count by continent.

GDAT contains more dams in all six continents of the world (Fig. 14) compared to GRanD and AQUASTAT. GOODD contains 10 percent more dam observations than GDAT, most of which are in Asia. GeoDAR has fewer observations than GDAT globally, but it has more coverage in North America, Europe, and Oceania. This suggests existing global dam databases may be biased toward developed countries and may be underestimating the existence of dams in lower-income and developing countries. In contrast, GDAT significantly improves data coverage in South America, Africa, and Asia, where multiple new primary sources were consulted in the data collection process. Compared to AQUASTAT, countries with significantly higher dam counts in GDAT include India (1,440 more), China (446 more), Iran (241 more), Brazil (4,863 more), South Africa (4,340 more), and various countries in the Southeast Asian region. For instance, Myanmar, Thailand, Laos, Cambodia, and Vietnam collectively have 575 more dams in GDAT than in AQUASTAT. Many of these countries are in the Global South, where economic development has given rise to rapid dam construction, yet dam-related data is often sparse and difficult to use. As such, GDAT is one of the most comprehensive geo-referenced global dam databases to date, with significant improvements in the coverage of the Global South.

As a further step in cross validation, we assess the spatial overlap between GDAT, GOODD, and GeoDAR. GOODD and GeoDAR are suitable datasets for cross-comparison, because they are independent data collection efforts that draw from different sources and use different data validation methods. We cannot match and cross-check individual dams due to the lack of dam name and attribute information in GOODD and GeoDAR. Instead, we assess the extent to which points in the GDAT, GOODD, and GeoDAR databases fall within the same geographic areas, which may indicate overlaps in dam coverage. To do so, we draw a 0.1° buffer zone around dams in each dataset. We then took the buffer zones for GDAT and verified the percentage of dams in GOODD and GeoDAR that overlapped with the GDAT buffer zones. We repeated this procedure for the GOODD and GeoDAR buffer zones.

Table 3 shows the percentage of each dataset captured by the 0.1° buffer zones. Around 54% of GDAT dams are captured by the GOODD and GeoDAR buffer zones. Between 49–52% of GOODD dams are captured by GDAT and GeoDAR buffer zones, while between 60–70% of GeoDAR dams are captured by GOODD and GDAT buffer zones. The comparison reveals that a substantial subset of each dataset is unique, highlighting the need for coordinated data collection efforts to create a globally consistent and comprehensive dam database.

**Table 3:** Cross-comparison of datasets–Percentage of overlap of dam location within 0.1° buffer zones.

Buffer Zones (0.1°)	Dataset		
	GDAT	GOODD	GeoDAR
GDAT	100%	48.9%	63.9%
GOODD	53.6%	100%	70.0%
GeoDAR	54.0%	52.1%	100%

#### Catchments

We compare the catchment areas of GDAT dams to those of GOODD, which is another global dam dataset with algorithm-generated catchment. Validating our catchment areas against GOODD^22^ is the most appropriate because both use similar procedures. This includes snapping the dams onto a river network from HydroRivers and using PCRaster and elevation data to capture the drainage area upstream of each dam.

**Fig. 15:**
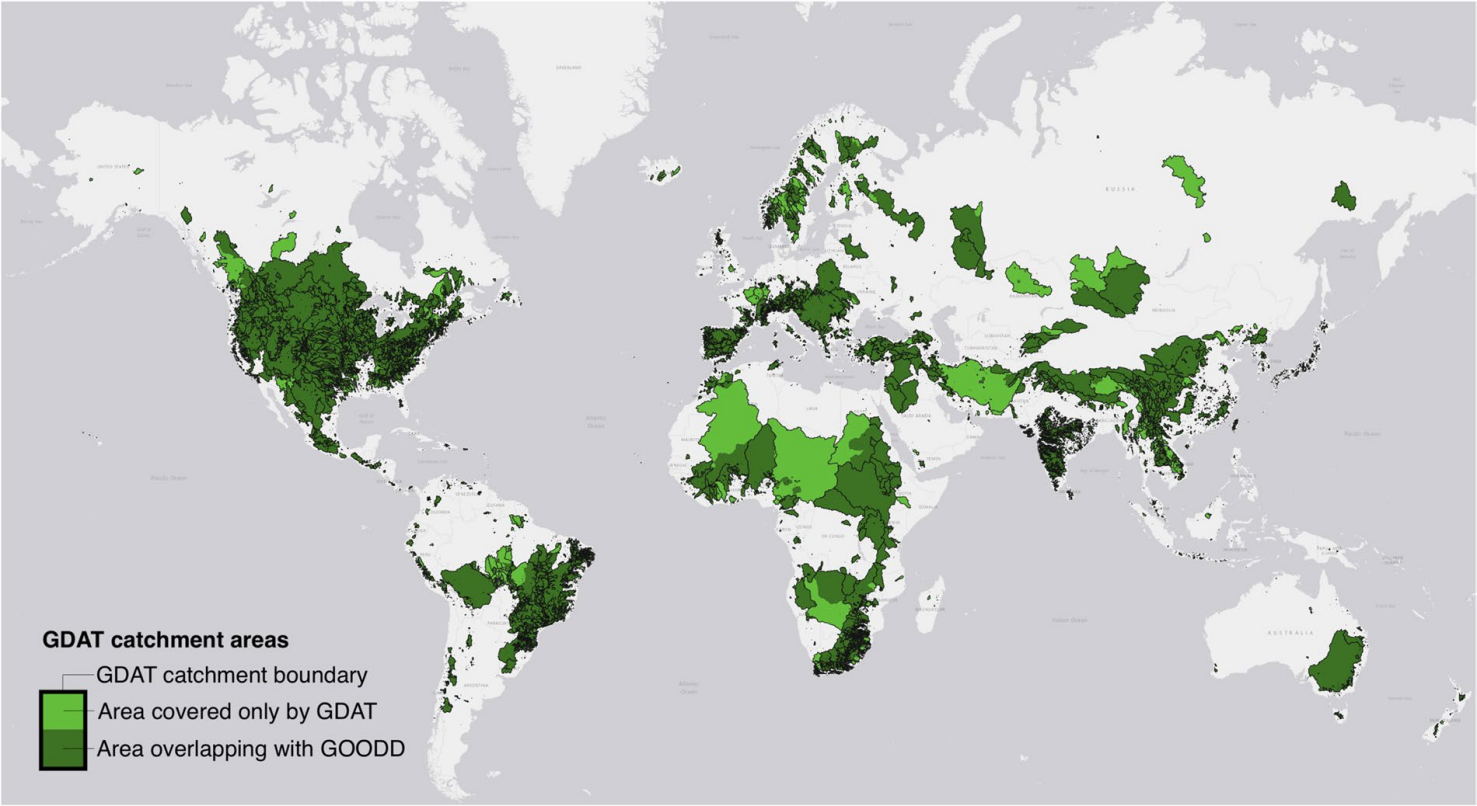
Comparison between GDAT and GOODD catchment areas. GDAT covers a total catchment areas of 44.88 million km^2^, while GOODD covers a total of 46.77 million km^2^. Catchment areas from GDAT and GOODD have 32.85 million km^2^ of overlap.

GDAT and GOODD are comparable in total catchment area coverage. GDAT catchment areas cover a total of 44.88 million km^2^, while GOODD catchment areas cover a total of 46.77 million km^2^. As shown in Fig. 15, GDAT and GOODD catchment areas overlap substantially. The overlapping area between the two datasets is 32.85 million km^2^, which constitutes 73.20% of GDAT catchments and 70.24% of GOODD catchments. Nevertheless, many non-overlapping areas in each dataset are due to extremely large catchment areas, especially in parts of Africa, the Middle East, Central Asia, and Eastern Europe, where few dams exist.

Within the overlapping areas, GDAT and GOODD catchments differ in size and extent. This is due to variations in regional dam counts between the two datasets. For instance, GOODD catchments cover more area in Southern China and Southeast Asia, but GDAT contains a higher density of dams in Southwestern China and Southeast Asia, making GDAT catchments smaller and more concentrated compared to GOODD catchments in this region. Given the lack of dam attributes in GOODD, we are unable to match catchment areas and compare them at an individual level. However, the differences between GDAT and GOODD catchment areas demonstrate the importance of consolidating datasets to ensure consistency.

### Region highlights

#### Africa

**Fig. 16:**
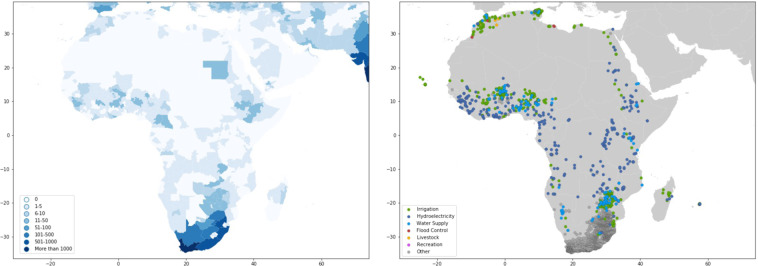
GDAT Africa – Number and main purpose of dams by location. Many major river basins throughout Africa are heavily dammed, such as the Nile, Western African rivers (Niger, Volta, Senegal), and Southern African rivers (Zambezi, Limpopo). South Africa shows a particularly high concentration of dams, in part due to better data accessibility.

Dams in Africa (Fig. 16) are concentrated in Sub-Saharan Western Africa (Niger river basin), Southern Africa, the Nile river basin, and Morocco. South Africa alone has published information for over 4,000 dams. Among the largest dams in Africa is the Aswan High Dam in Egypt, whose reservoir (Lake Nasser) can hold roughly 132 km^2^ of water, and the Akosombo Dam in Ghana, with a reservoir capacity of 48 km^2^ and covers more than 3 percent of Ghana’s land area. In addition to the dams on the continent itself, small dams are also present on the small island nations Cape Verde and Mauritius. Most dams in Africa are hydroelectricity, irrigation, or water supply dams.

The large number of dams shown in South Africa is mainly due to the availability of published data from the South African government (www.dws.gov.za/hydrology). For much of the continent, the predominant databases used were AQUASTAT and GRanD. Other than South Africa, the rest of the continent has a relatively small number and low density of dams compared to the rest of the world based on available data. Nevertheless, many large hydropower projects are currently being planned, including nearly 150 projects being tracked by International Rivers. Many large dams are funded by China, which, according to International Rivers, committed more than $3 billion to dam construction in Africa between 2001 and 2007^42^. Policy drivers such as China’s Belt and Road Initiative have been channeling more funds from China to large infrastructure projects in Africa, such as the Kafue Gorge Lower dam in Zambia, the Gilgel Gibe III Dam in Ethiopia, and a $5.8 billion hydropower project in Nigeria that involves four dams^43^.

#### Asia

**Fig. 17:**
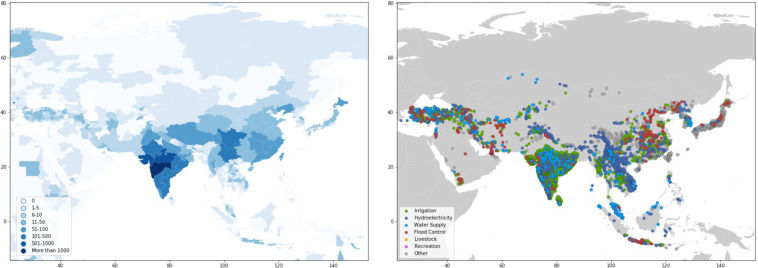
GDAT Asia – Number and main purpose of dams by location. For Asia, high concentrations of dams exist in major river basins such as the Mekong, Yangtze, Yellow, and Tigris/Euphrates, as well as in many regions such as the Indian subcontinent, Indochinese peninsula, Java, Japan, the Korean peninsula, and the Anatolian peninsula. Many of these regions are home to rapidly developing economies with a high population density.

Most GDAT dams in Asia are concentrated in India and Eastern China (Fig. 17). India alone has over 4,000 dams, while China has more than 1,000. Japan, Turkey, Iran, and Southeast Asia each have more than 500 dams. The largest concentrations of dams in India can be found in the central-western part of the country. While most dams in India are small-scale irrigation dams, significant numbers of hydroelectric and water-supply dams are scattered throughout the central-western parts of the country. The northern and northwestern parts of the country (Rajasthan, Delhi, Uttar Pradesh, Bihar), where the elevation is flatter and lower than the central and southern regions, have a significantly lower density of dams. There are very few dams in the Indo-Gangetic Plain in the northeastern part of the country, as well as in the Thar Desert in Rajasthan.

Most dams in China are located in Sichuan and Yunnan, two provinces with high elevations and several fast-flowing rivers. Southern China and the Yangtze basin have more dams than Northern China, and dams are mostly concentrated in mountainous areas–there are no dams in the North China Plain. The country has many flood control dams relative to surrounding countries and regions.

There is an especially high density of dams on the watersheds of the upper Yangtze, upper Mekong, and upper Salween rivers, which collectively form the Three Parallel Rivers biosphere reserve. Many of these dams are planned, under construction, or canceled. Being cross-border rivers, dams on the Yangtze, Mekong, and Salween rivers are extensively tracked by international organizations because of the geopolitical implications of dam construction, which partly explains the larger number of dams shown in these areas. Fear among Southeast Asian countries of China’s upstream dam-building activities have prevailed, despite regional organizations’ best efforts, due to an unequal power dynamic^44^. Further proposals to construct large hydropower dams on China’s southwestern rivers have caused concern among downstream neighbors^45^.

There is a high concentration of dams in peninsular Malaysia and on the island of Java, Indonesia. Turkey and Iran have the highest numbers and concentrations of dams in the Middle East, especially because of the aggressive dam-construction policies implemented by both countries. The highest density of dams in Iran is found in the northwestern part of the country around Lake Urmia, an endorheic lake that has shrunken significantly due to dam construction^46^.

Because of the upstream location of Turkey and Iran on the Tigris and Euphrates river basins, respectively, dam construction in both countries has raised geopolitical concerns by neighboring downstream countries such as Iraq and Syria. Turkey, which has constructed dams on the Tigris and Euphrates basins as early as the 1950s, has proposed more large-dam projects that are expected to significantly reduce the flow of major rivers into Syria and Iraq. The country has also engaged in water politics against its downstream neighbors^47^. As in Africa, the damming of rivers has caused wetlands and previously fertile agricultural lands to dry up, displacing people and triggering geopolitical tensions^48^.

#### Oceania

**Fig. 18:**
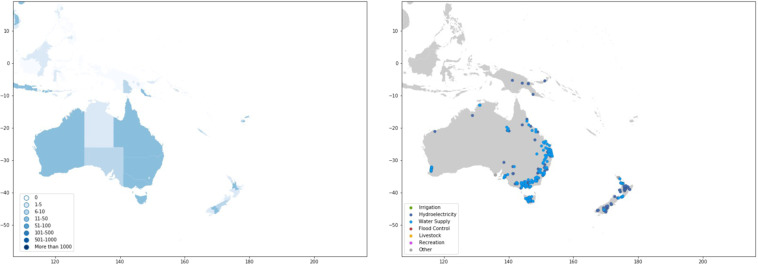
GDAT Oceania – Number and main purpose of dams by location. On the Australian mainland, dams are mostly concentrated in the Eastern region, along the Great Dividing Range. Significant numbers of dams are also present in Tasmania, as well as on the islands of New Zealand.

There are more than 500 dams in Australia and roughly 70 in New Zealand (Fig. 18). Dams in Australia are mostly concentrated in the coastal regions of the continent and on Tasmania, while dams in New Zealand are relatively evenly distributed between the North and South Island. A few Pacific island countries (Fiji, Samoa) and Papua New Guinea also contain a small number of dams. Dams in Oceania are used mostly for hydroelectricity and water supply and are no bigger than a few thousand cubic meters in reservoir capacity.

#### Europe

**Fig. 19:**
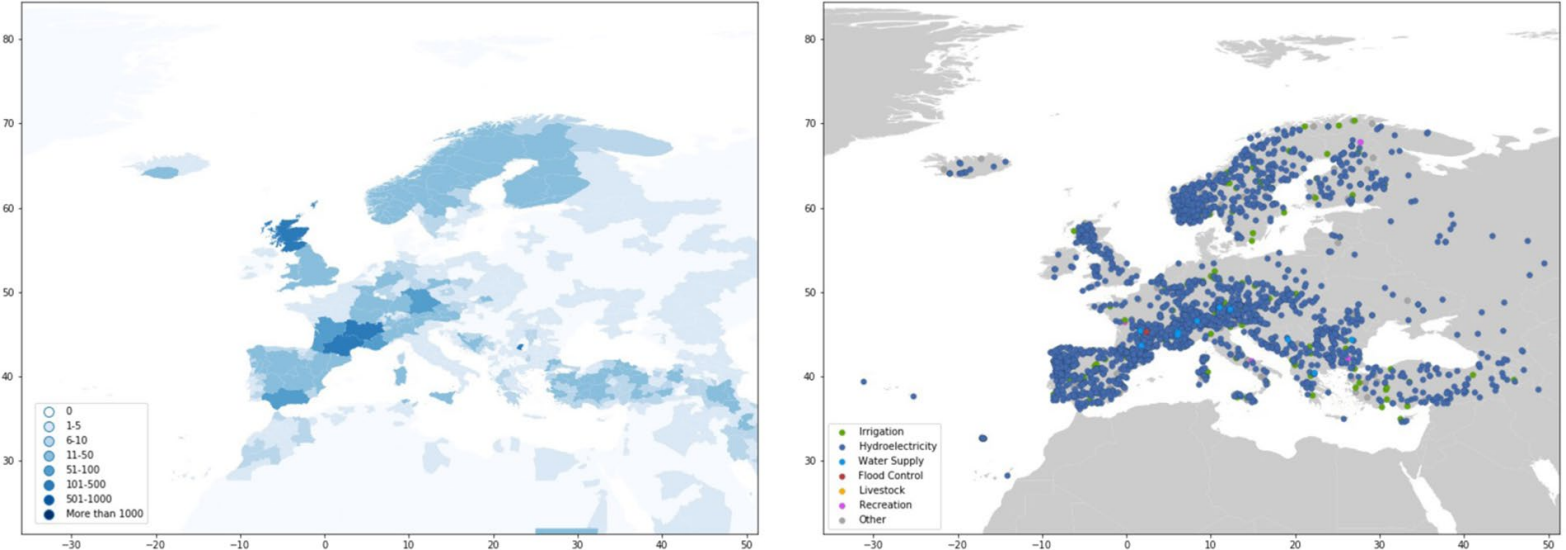
GDAT Europe – Number and main purpose of dams by location. For Europe, the high frequency of hydroelectric dams is due to the large number of observations supplied by the World Resources Institute Global Power Plant database. Significant concentrations of dams are present in the Iberian peninsula, Southern France, the Alps, Scotland, and Southern Norway.

In Europe, the highest concentration of dams can be found in southern France, Scotland, southern Norway, the Iberian Peninsula, and the Alpine regions of Central Europe (Fig. 19). On average, dams in Western Europe tend to be smaller in reservoir capacity than those in Eastern Europe and Russia, even though they are more numerous. While the rate of dam construction was high in the past two centuries, it has significantly slowed in the present day.

#### North America

**Fig. 20:**
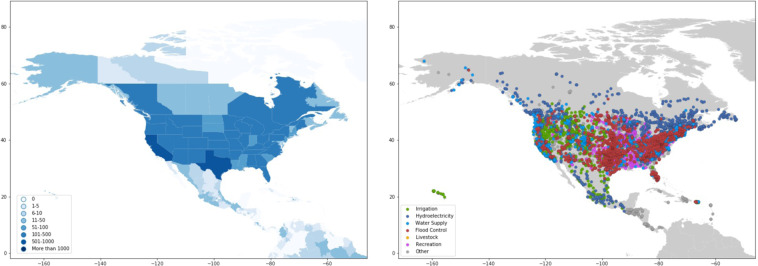
GDAT North America – Number and main purpose of dams by location. Data for the United States mainly come from USGS, with small dikes and non-dam structures removed from the database. In Canada, most dams are concentrated closer to the southern border, with Quebec having a high concentration of hydroelectric dams. Central Mexico also contains a high concentration of dams.

The United States and Canada have some of the highest concentration of dams in the world (Fig. 20). After removing dikes and other non-dam structures, we obtain 7,039 dams from the USGS (nationalmap.gov). The GDAT database lists more than 600 dams in Canada, most of which are concentrated in Ontario, Quebec, and British Columbia. Canada boasts some of the largest hydroelectric dams in the world and is among the world’s largest hydropower generators.

In the United States, California and Texas stand out as having the largest dam counts, while most states have more than 100. Although there are many large dams in the United States, the vast majority are small. The USGS data also contain information for dikes and small-river hydroelectric plants that do not necessarily involve dams. To ensure consistency, we removed dikes and non-dam structures from the USGS database.

Dam construction in the United States has taken place since the early nineteenth century to improve navigation on major rivers and provide electricity. During that time, the American public treated water as a commodity that needed to be improved through channeling and establishing waterworks. The utilitarian mindset toward water resources persisted throughout the Progressive Era under then-president Theodore Roosevelt, who believed that natural resources needed to be used efficiently to provide the greatest good for the greatest number of people^49^. In subsequent decades, the United States constructed many dams to power its industrial economy. Many dams in the United States are now old and have reached or exceeded their design life, leading to a need for dam removal^50^.

#### South America

**Fig. 21:**
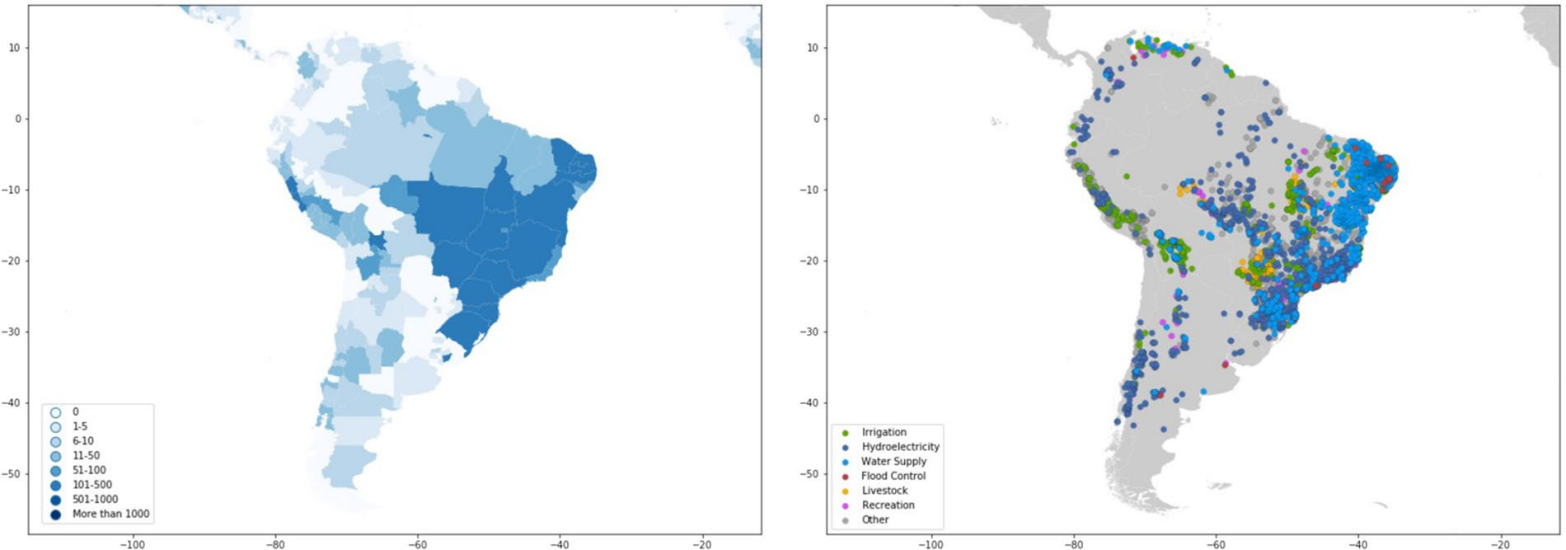
GDAT South America – Number and main purpose of dams by location. The Amazon basin is home to several large dams, while the highest concentration of dams can be found in the eastern and southeastern regions of the country. The Andes also contain many dams, particularly in Peru and central Chile.

In South America (Fig. 21), Brazil has over 5,200 dams (if dikes were included, Brazil would have the largest recorded number of dams and dikes). Most dams in Brazil are located along the east coast around the population centers of São Paulo, Rio de Janeiro, and Belo Horizonte. Most dams in Brazil are used for hydroelectricity and water supply. Brazil depends on hydroelectricity for over 60 percent of its electric power supply, and a recent boom in the small hydropower sector has put the country on track toward an energy surplus^51^.

However, Brazil’s hydropower construction boom in the Amazon basin has resulted in the destruction and inundation of large rainforest areas. For example, the Belo Monte Dam, the world’s fourth-largest hydroelectric project, flooded 260 square miles of forested lowlands and displaced more than 20,000 indigenous people^52^. In response to intense local and international resistance from environmentalists and indigenous groups, the Brazilian government recently announced a major shift away from its policy of building mega-dams in the Amazon basin^53^.

Although the construction of large mega-dams may ramp down, Brazil is still experiencing a rapid expansion of small hydroelectric plants. Massive investments by the private sector after 1995 were stimulated by economic incentives and new regulations within the energy market, resulting in hundreds of new small dams. A recent study by researchers at the University of Washington estimates that 1,007 small hydroelectric plants are currently operating in Brazil^5^. An additional 35 are under construction, and 156 are approved and awaiting final licensing. Their research reports that 33 small hydropower plants have been constructed per year on average from 2001 to 2016, a growth rate 14 times as fast as that witnessed in the 1990s.

Other Andean countries (Bolivia, Chile) also have significant numbers of dams, more than 600 of which are located in the Peruvian Andes and used mostly for irrigation and hydroelectricity. In other parts of the Andes, most dams are used for hydroelectricity, water supply, irrigation, and recreation. The Guri Dam in Venezuela, a hydroelectric dam with a reservoir volume of 135 km^3^, ranks among the largest dams in South America and in the world. According to AQUASTAT, Uruguay is home to the second largest number of dams in South America, following Brazil. However, after spot-searching individual listings, the authors could only find location data for 4 of the 878 dams and reservoirs included. Additional research will be needed to confirm the validity and physical location of these structures.

## Data Availability

Code^54^ for replicating results in this article is publicly available on Zenodo (10.5281/zenodo.6784716). We use Python (versions 3.6 and 3.7), Stata MP (version 15.1), Google Earth Engine (https://earthengine.google.com) to obtain dam catchment data and conduct analysis.

## References

[1] IEA. Renewables 2020. Tech. Rep., IEA (2020).

[2] IEA. Electricity Information: Overview. Tech. Rep., IEA (2021).

[3] Gernaat DEHJ, Bogaart PW, van Vuuren DP, Biemans H, Niessink R (2017). High-resolution assessment of global technical and economic hydropower potential. Nature Energy.

[4] Zarfl C, Lumsdon AE, Berlekamp J, Tydecks L, Tockner K (2015). A global boom in hydropower dam construction. Aquat. Sci..

[5] Couto TBA, Olden JD (2018). Global proliferation of small hydropower plants - science and policy. Front. Ecol. Environ..

[6] World Commission on Dams. *Dams and Development: A New Framework for Decision-making: Report of the World Commission on Dams* (Earthscan, 2000).

[7] Zhang, A. T. Within but without: Involuntary displacement and economic development. Preprint at 10.2139/ssrn.3358089 (2018).

[8] Wang P, Lassoie JP, Dong S, Morreale SJ (2013). A framework for social impact analysis of large dams: a case study of cascading dams on the Upper-Mekong River, China. J. Environ. Manage..

[9] Haddeland I (2014). Global water resources affected by human interventions and climate change. Proc. Natl. Acad. Sci. USA.

[10] Schindler DW, Donahue WF (2006). An impending water crisis in Canada’s western prairie provinces. Proc. Natl. Acad. Sci. USA.

[11] Noori, R. *et al*. Anthropogenic depletion of Iran’s aquifers. *Proc. Natl. Acad. Sci. USA* **118** (2021). 10.1073/pnas.2024221118 PMC8237601 34161268

[12] Müller MF, Yoon J, Gorelick SM, Avisse N, Tilmant A (2016). Impact of the Syrian refugee crisis on land use and transboundary freshwater resources. Proc. Natl. Acad. Sci. USA.

[13] Del Bene D, Scheidel A, Temper L (2018). More dams, more violence? A global analysis on resistances and repression around conflictive dams through co-produced knowledge. Sustainability Sci..

[14] Fearnside PM (2016). Environmental and Social Impacts of Hydroelectric Dams in Brazilian Amazonia: Implications for the Aluminum Industry. World Dev..

[15] Maavara T (2015). Global phosphorus retention by river damming. Proc. Natl. Acad. Sci. USA.

[16] Zarfl C (2019). Future large hydropower dams impact global freshwater megafauna. Sci. Rep..

[17] Ziv G, Baran E, Nam S, Rodrguez-Iturbe I, Levin SA (2012). Trading-off fish biodiversity, food security, and hydropower in the Mekong River Basin. Proc. Natl. Acad. Sci. USA.

[18] Barbarossa V (2020). Impacts of current and future large dams on the geographic range connectivity of freshwater fish worldwide. Proc. Natl. Acad. Sci. USA.

[19] Winemiller KO (2016). Balancing hydropower and biodiversity in the Amazon, Congo, and Mekong. Science.

[20] Dugan PJ (2010). Fish migration, dams, and loss of ecosystem services in the Mekong basin. Ambio.

[21] Wohlfahrt G, Tomelleri E, Hammerle A (2021). The albedo-climate penalty of hydropower reservoirs. Nat Energy.

[22] Mulligan M, van Soesbergen A, Sáenz L (2020). GOODD, a global dataset of more than 38,000 georeferenced dams. Sci Data.

[23] Wang J (2022). GeoDAR: georeferenced global dams and reservoirs dataset for bridging attributes and geolocations. Earth System Science Data.

[24] Zhang, A. T., Urpelainen, J. & Schlenker, W. Power of the River: Introducing the Global Dam Tracker (GDAT). Tech. Rep., Center on Global Energy Policy (2018).

[25] Lehner B (2011). High-resolution mapping of the world’s reservoirs and dams for sustainable river-flow management. Front. Ecol. Environ..

[26] Byers, L., *et al*. A Global Database of Power Plants. Tech. Rep., World Resources Institute (2018).

[27] Lehner B, Grill G (2013). Global river hydrography and network routing: baseline data and new approaches to study the world’s large river systems. Hydrol. Process..

[28] Danielson, J. J. & Gesch, D. B. Global Multi-Resolution Terrain Elevation Data 2010 (GMTED2010). Tech. Rep., US Geological Survey (2011).

[29] Karssenberg D, Schmitz O, Salamon P, de Jong K, Bierkens MF (2010). A software framework for construction of process-based stochastic spatio-temporal models and data assimilation. Environmental Modelling & Software.

[30] Pekel J-F, Cottam A, Gorelick N, Belward AS (2016). High-resolution mapping of global surface water and its long-term changes. Nature.

[31] Linke S (2019). Global hydro-environmental sub-basin and river reach characteristics at high spatial resolution. Scientific data.

[32] Gillies, S. The shapely user manual. https://pypi.org/project/Shapely (2013).

[33] Lehner, B. *HydroRivers Technical Documentation Version 1.0* (2019).

[34] Hansen BE (2001). The new econometrics of structural change: dating breaks in us labour productivity. Journal of Economic perspectives.

[35] Chu C-SJ, Stinchcombe M, White H (1996). Monitoring Structural Change. Econometrica.

[36] Piehl AM, Cooper SJ, Braga AA, Kennedy DM (2003). Testing for structural breaks in the evaluation of programs. Rev. Econ. Stat..

[37] Davies RB (1987). Hypothesis testing when a nuisance parameter is present only under the alternative. Biometrika.

[38] Andrews, D. W. Tests for parameter instability and structural change with unknown change point. *Econometrica: Journal of the Econometric Society* 821–856 (1993).

[39] Kim H-J, Siegmund D (1989). The likelihood ratio test for a change-point in simple linear regression. Biometrika.

[40] Quandt RE (1960). Tests of the hypothesis that a linear regression system obeys two separate regimes. Journal of the American statistical Association.

[41] Zhang AT, Gu VX (2023). Zenodo.

[42] Hathaway, T. What is Driving Dams in Africa? Tech. Rep., International Rivers (2010).

[43] Mongalvy, S., Malingha, D. & Sguazzin, A. Nigeria to Start Building $5.8 Billion Power Plant in 2018. *Bloomberg News* (2018).

[44] Wong, C. China and the Mekong: The Floodgates of Power. The Diplomat Accessed: 2022-5-27 (2016).

[45] Walker, B. China gives green-light to new era of mega-dams. China Dialogue. Accessed: 2022-5-27 (2013).

[46] Rahimi, A. & Breuste, J. Why is Lake Urmia Drying up? Prognostic Modeling With Land-Use Data and Artificial Neural Network. *Front. Environ. Sci*. **9** (2021).

[47] Jongerden J (2010). Dams and politics in turkey: Utilizing water, developing conflict. Middle East Pol..

[48] Levkowitz, J. Iraq wilting: How creeping drought could cause the next crisis. Middle East Institute. Accessed: 2022-5-27 (2018).

[49] Billington, D. P., Jackson, D. C. & Melosi, M. V. *The History of Large Federal Dams: Planning, Design, and Construction in the Era of Big Dams* (2005).

[50] Ho M (2017). The future role of dams in the United States of America. Water Resour. Res..

[51] EIA. Country Analysis Brief - Brazil. EIA. Accessed: 2022-5-27 (2022).

[52] Amazon Watch. Fact sheet: The Belo Monte Dam. Amazon Watch. Accessed: 2023-2-6 (2011).

[53] Watts, J. Brazil raises hopes of a retreat from new mega-dam construction. *The Guardian* (2018).

[54] Zhang AT, Gu VX (2023). Zenodo.

